# Advances in Protozoan Epigenetic Targets and Their Inhibitors for the Development of New Potential Drugs

**DOI:** 10.3390/ph16040543

**Published:** 2023-04-04

**Authors:** Carlos Gaona-López, Lenci K. Vazquez-Jimenez, Alonzo Gonzalez-Gonzalez, Timoteo Delgado-Maldonado, Eyrá Ortiz-Pérez, Benjamín Nogueda-Torres, Adriana Moreno-Rodríguez, Karina Vázquez, Emma Saavedra, Gildardo Rivera

**Affiliations:** 1 Laboratorio de Biotecnología Farmacéutica, Centro de Biotecnología Genómica, Instituto Politécnico Nacional, Reynosa 88710, Mexico; 2 Departamento de Parasitología, Escuela Nacional de Ciencias Biológicas, Instituto Politécnico Nacional, Mexico City 11340, Mexico; 3 Laboratorio de Estudios Epidemiológicos, Clínicos, Diseños Experimentales e Investigación, Facultad de Ciencias Químicas, Universidad Autónoma “Benito Juárez” de Oaxaca, Avenida Universidad S/N, Ex Hacienda Cinco Señores, Oaxaca 68120, Mexico; 4 Facultad de Medicina Veterinaria y Zootecnia, Universidad Autónoma de Nuevo León, Francisco Villa 20, General Escobedo 66054, Mexico; 5 Departamento de Bioquímica, Instituto Nacional de Cardiología Ignacio Chávez, Mexico City 14080, Mexico

**Keywords:** epigenetic targets, drugs, inhibitors, protozoa

## Abstract

Protozoan parasite diseases cause significant mortality and morbidity worldwide. Factors such as climate change, extreme poverty, migration, and a lack of life opportunities lead to the propagation of diseases classified as tropical or non-endemic. Although there are several drugs to combat parasitic diseases, strains resistant to routinely used drugs have been reported. In addition, many first-line drugs have adverse effects ranging from mild to severe, including potential carcinogenic effects. Therefore, new lead compounds are needed to combat these parasites. Although little has been studied regarding the epigenetic mechanisms in lower eukaryotes, it is believed that epigenetics plays an essential role in vital aspects of the organism, from controlling the life cycle to the expression of genes involved in pathogenicity. Therefore, using epigenetic targets to combat these parasites is foreseen as an area with great potential for development. This review summarizes the main known epigenetic mechanisms and their potential as therapeutics for a group of medically important protozoal parasites. Different epigenetic mechanisms are discussed, highlighting those that can be used for drug repositioning, such as histone post-translational modifications (HPTMs). Exclusive parasite targets are also emphasized, including the base J and DNA 6 mA. These two categories have the greatest potential for developing drugs to treat or eradicate these diseases.

## 1. Introduction

Parasitic diseases represent a worldwide public health problem that causes many deaths, especially in developing countries. Furthermore, climate change and migration, among other factors, increase the incidence of these diseases in non-endemic countries. Although there are drugs to treat the infections caused by many of these parasites, they have drawbacks due to mild, moderate, or severe adverse effects (dizziness, headaches, and even carcinogenic effects) and increased parasite drug resistance coupled with the lack of therapeutic adherence due to the various adverse effects listed. In some cases, drug treatment has a high cost or is unavailable. Therefore, developing new drugs to combat the different diseases caused by these parasites is imperative [1,2,3].

A promising strategy for controlling gene expression in lower eukaryotes has been hypothesized. Such a strategy would regulate the expression of genes involved in pathogenicity, differentiation in the life cycle, and other vital aspects of the pathogen. Therefore, identifying new epigenetic targets would contribute immensely to controlling or even eradicating these diseases [4,5,6,7].

The term “epigenetics” has been discussed to generate a standardized definition. In the middle of the last century, Waddington considered that “an epigenetic trait is a stably heritable phenotype resulting from changes in a chromosome without alterations in the DNA sequence” [8].

In this context, an “epigenetic target” would involve the actors in charge of modulating gene expression without altering the DNA sequence. These so-called epigenetic enzymes, classified as writers (enzymes responsible for methylating both DNA and histones and other enzymes responsible for adding functional groups on histones), erasers (enzymes responsible for removing functional groups on histones), and readers (bromodomain proteins) are the key actors. It is also important to mention that non-coding RNA are used as therapeutic targets because of their ability to modulate transcription [9,10,11]. Lastly, the term epidrugs has recently emerged to refer to drugs targeting different epigenetic mechanisms. These epidrugs can be very useful in combating cardiovascular diseases, disorders of a metabolic and neurological nature, and cancer, which are associated with an abnormality of regulation of epigenetic mechanisms [12,13,14].

In this literature review, the main objective was to summarize and analyze the epigenetic mechanisms that can be used as therapeutic targets and the advances in the discovery and development of inhibitors against seven parasites: *Trypanosoma cruzi* (*T. cruzi*), *Trypanosoma brucei* (*T. brucei*), *Leishmania* spp., *Entamoeba histolytica* (*E. histolytica*), *Giardia lamblia* (*G. lamblia*), *Toxoplasma gondii* (*T. gondii*), and *Trichomonas vaginalis* (*T. vaginalis*) as an alternative to obtaining new antiprotozoal drugs. Each section shows the main characteristics of each parasite disease, its prevalence, impact on public health, and pharmacological treatment (doses and side effects). Different epigenetic mechanisms of organisms are described and discussed. Finally, previous inhibitors in each epigenetic target are described. The activity values of the reference drugs were taken from the specialized literature. Half-maximal inhibitory concentration (IC_50_), half-maximal cytotoxic concentration (CC_50_), half-maximal effective concentration (EC_50_), and selective index (SI) values of the evaluated compounds were taken from experiments carried out in different research, corresponding to phenotypic assays. The search was conducted in the PubMed, ScienceDirect, and Google Scholar databases using the keywords: epigenetics, epigenetic target, drugs, protozoa, and inhibitors.

## 2. *Trypanosoma cruzi*

*T. cruzi* is a flagellated unicellular protozoon belonging to the Kinetoplastida class. It is the etiological agent of Chagas disease, also known as American trypanosomiasis. *T. cruzi* has a single flagellum and a distinctive single mitochondrion, which harbors a significant amount of DNA in a kinetoplast structure [15,16]. This parasite is classified into seven discrete typing units (DTUs), *Tc*I-*Tc*VI and *Tc*bat, based on its genetic and biological diversity. Each presents different clinical manifestations and drug sensitivity. It is worth mentioning that trypanosomiasis is classified as one of the main “Neglected Tropical Diseases” (NTD) by the World Health Organization (WHO, 2023) [17,18,19,20].

**Figure 1:**
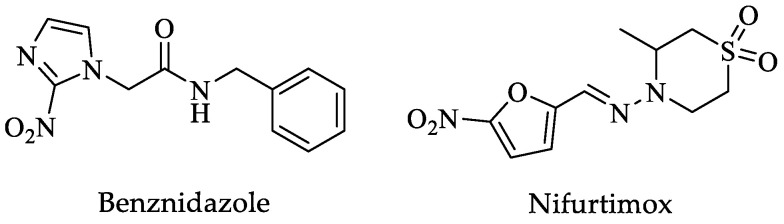
Chemical structures of the two drugs used against Chagas disease.

Around seven million cases of Chagas disease are reported worldwide, and about 75 million people are at risk. It is also estimated that 30% of the infected population is prone to complications from infection, mainly cardiomyopathy, megaesophagus, and megacolon, which can be lethal. This disease is endemic in twenty-one Latin American countries [20], and according to the World Health Organization (WHO), more than 10,000 deaths per year are estimated. The drugs for treating this parasitosis are benznidazole (Bzn) and nifurtimox (Nfx) (Figure 1). These drugs are effective in treating this disease if administered early after infection, but their effectiveness decreases in adults and the chronic phase of the disease. Furthermore, important side effects have been reported; thus, new drugs are needed to treat this parasitic disease (Table 1) [20,21,22,23,24,25,26,27,28,29].

The epigenetic aspects that regulate gene expression in *T. cruzi* are unclear. Nevertheless, different histone acetylation states have been reported at various stages of this parasite’s life cycle. Therefore, post-translational modifications can play an essential role in regulating vital aspects of this organism.

### Histone Post-Translational Modifications (HPTMs)

HPTMs consist of adding chemical groups to certain amino acid residues of histones, mainly at the amino-terminal end. They function as highly specific signals that open or close chromatin domains, thus modulating their transcriptionally active or silenced states [33].

One of the most well-known HPTMs is acetylation, a dynamic process controlled by two opposite enzymes: histone acetyltransferase (HAT), which catalyzes the transfer of acetyl groups from acetyl-CoA to amino-terminal lysines of histones; and histone deacetylase (HDAC), which eliminates these acetyl groups [34].

In the case of *T. cruzi* and other trypanosomatids, the existence of different states of histone acetylation in different cell cycle stages is well documented. Therefore, it is tempting to think that epigenetic control could be decisive for the cell cycle of these organisms [35].

**Figure 2:**
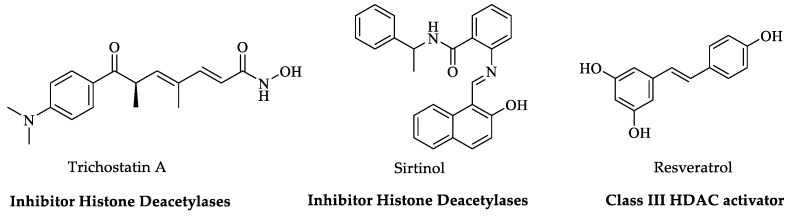
Structure of compounds with epigenetic effects against *T. cruzi*.

In 2017, Campo reported two drugs that potentially inhibit the HDAC of *T. cruzi*: trichostatin A and sirtinol (Figure 2). Both are known for their inhibitory effect on the different HDACs, and resveratrol (Figure 2), a known class III HDAC activator. These compounds were tested in the different life cycle stages of *T. cruzi* [36,37]. The results indicated that both HDAC inhibitors caused histone hyperacetylation, especially of H4 and H2B. The effect was dependent on the inhibitor concentration used. Additionally, sirtinol caused an increase in the level of acetylation of histone H3. The effects were observed in trypomastigotes and epimastigotes, although the former presented a higher acetylation level in their histones [36].

As expected, resveratrol decreased the acetylation levels of different histones. These analyses showed that all three compounds greatly affected histone acetylation, potentially by regulating HDAC activity in *T. cruzi* [36]. However, the inhibitors did not affect *T. cruzi* growth or replication. In contrast, epimastigotes treated with resveratrol showed a high inhibitory effect on the replication rate with an IC_50_ of 250 μM [36]. On the other hand, HDAC inhibitors affect the life cycle of other parasites, generally promoting or inhibiting the differentiation process. In the case of *T. cruzi*, trichostatin A and sirtinol reduced the differentiation process from epimastigotes (replicative form) to trypomastigotes (infective form) by 20%, while resveratrol caused a 30% increase in differentiation [36]. Finally, this study analyzed the effect of the three compounds on the infectivity of *T. cruzi*. The data showed that only resveratrol reduced the percentage of live parasites in infected Vero cells, with an IC_50_ of 50.3 μM. These results pinpoint resveratrol as a possible drug to combat trypanosomiasis, targeting the trypanosome’s HDACs [36,37].

Histone acetylation is usually linked to transcriptional activation, whereas low histone acetyl group content restores heterochromatin structure, contrary to gene activation. Additionally, acetylation moieties on histones serve as recruitment points for transcription regulators [38].

Bromodomain proteins are “readers” of acetylated lysines on histone tails. They present a pocket that recognizes acetylated lysine residues, giving rise to a protein–protein interaction between histones and bromodomain proteins. This interaction leads to the recruitment of chromatin remodeling complexes and general transcription factors [39,40].

A few years ago, a series of drugs, denominated as bromo, and extra terminal (BET) inhibitors, were described that reversibly bind to proteins with BET domains, specifically binding to the acetyl group recognition pocket. These BET inhibitors prevent the protein–protein interaction between bromodomain proteins and acetylated histones, thus precluding the binding of chromatin remodeling complexes and/or transcription factors, thereby inhibiting gene expression [39,40,41,42].

Only seven bromodomain proteins have been identified in *T. cruzi* (*Tc*BDF 1–7). This information was obtained through in silico analysis using the specialized database TriTrypDB. On the one hand, it has been reported that *Tc*BDF1 is present throughout the trypanosome life cycle. This protein, immunolocalized in the glycosome, is mostly expressed in the trypomastigote stage. Likewise, it was reported that overexpression of *Tc*BDF1 is detrimental to the growth and differentiation of epimastigotes. Additionally, overexpression of a mutant *Tc*BDF1 negatively affected the infectivity of trypomastigotes [42,43].

On the other hand, using Western blot assays, Villanova et al. showed that *Tc*BDF2 levels increased significantly in response to exposure to UV radiation in *T. cruzi*, suggesting that it forms part of a chromatin remodeling complex. Additionally, it was demonstrated with immunofluorescence and electronic microscopy analyses that such a protein is present within the nucleus during the different stages of the *T. cruzi* life cycle. Finally, by far-Western blot and pull-down assays, its interaction with H2B and H4 was demonstrated [44].

In the case of *Tc*BDF3, it binds to α-tubulin and is cyto-localized in the flagellum and the flagellar pocket. It is worth mentioning that this protein is essential for growth in the epimastigote stage, possibly modulating the microtubule cytoskeleton [42,43,44,45]. There is currently no information on the functions and cell locations of *Tc*BDF4 and *Tc*BDF5, while *Tc*BDF6 and *Tc*BDF7 have only been identified by in silico analysis.

**Figure 3:**
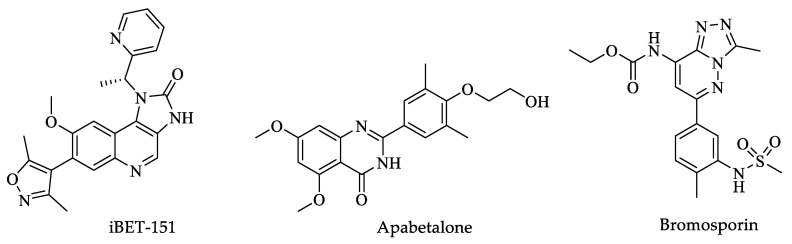
Structure of compounds with *Tc*BDF2 inhibitory activity of *T. cruzi*.

According to the aforementioned findings, *Tc*BDF2, the only one of these proteins immunolocalized in the nucleus, is an ideal candidate for an epigenetic therapeutic target because it presents unique characteristics not found in their mammalian counterparts, such as low identity sequence, a different inner core, and few conserved amino acids in the hydrophobic pocket [46]. Therefore, developing compounds with the inhibitory activity of this protein should be considered a priority [42,44]. An additional study by Pezza et al. with the help of CRISPR gene editing demonstrated that the *Tc*BDF2 protein is essential for the viability of *T. cruzi* within biological processes in which it is involved, such as regulating infectivity in the amastigote multiplication process and during metacyclogenesis. Finally, these authors reported that *Tc*BDF2 is susceptible to human inhibitors such as iBET-151, apabetalone (RVX-208), and bromosporin (BSP) (Figure 3). The use of mutated *Tc*BDF2 and far-Western blotting led to the hypothesis that these last inhibitors function by competing with the ligand for the hydrophobic pocket, making this protein a potential druggable target against *T. cruzi* [46].

## 3. *Trypanosoma brucei*

*T. brucei* is a diploid protozoan parasite which is the etiological agent of human African trypanosomiasis, or sleeping sickness, an endemic NTD of sub-Saharan Africa. There are two subspecies; each one varies in the rate of disease progression. The slowly progressive form is caused by the subspecies *T. brucei gambiense*, which is endemic in west and central Africa. It accounts for more than 90% of diagnosed infections. The rapid progressive form is caused by the subspecies *T. brucei rhodesiense*, which is endemic in east and south Africa. It accounts for less than 10% of the current cases of infection but is the most lethal due to its rapid evolution to the neurological phase [47,48]. Due to efforts made in recent decades to tackle the infection, the number of people infected has decreased, with about 21,000 cases in 2012. Despite this advancement, the overpopulation of the sub-Saharan Africa region causes more than 70 million people to be at risk of contracting this disease [49,50].

**Figure 4:**
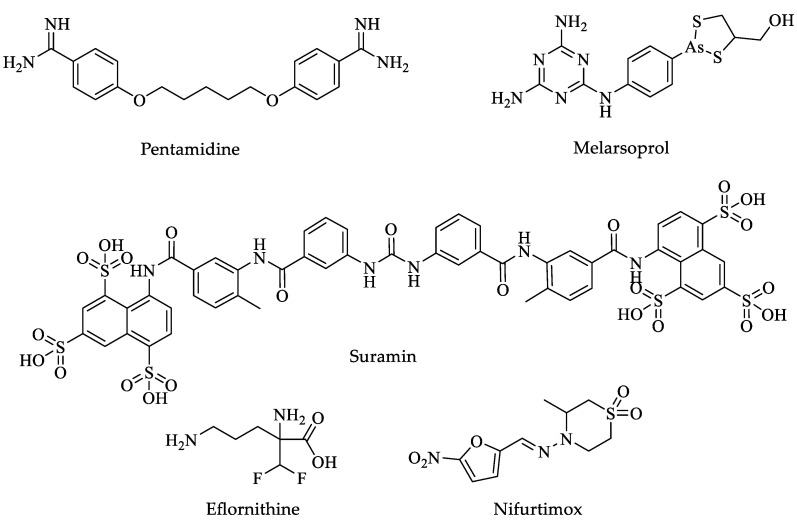
First and second-line drugs used against *T. brucei*.

There are only five drugs in routine use for human African trypanosomiasis treatment (Table 2). Pentamidine and suramin (Figure 4) are used to combat the first phase of the infection, while melarsoprol, eflornithine, and nifurtimox (Figure 4) are commonly used to treat the neurological phase. During this second phase, the drugs must cross the blood–brain barrier (BBB) but have the drawback of being more toxic [51,52,53,54,55,56,57,58].

All drugs that combat this disease cause severe and irreversible collateral effects, such as anemia, leucopenia, nephrotoxicity, peripheral neuropathy, and more [58]. Additionally, resistance of *T. brucei* to the most widely used drugs has been reported. Therefore, there is a constant search for specific and highly effective drugs against this parasite to treat and eventually eradicate this disease [51,52,53,54].

The epigenetic mechanisms responsible for regulating gene expression in *T. brucei* vary from HPTMs to using nitrogenous bases characteristic of the genus. An example of the complexity of the epigenetic mechanisms that regulate gene expression in this parasite is the remodeling of the variable surface glycoproteins (VSGs).

### 3.1. Variable Surface Glycoprotein (VSG)

*T. brucei*, like other parasitic protists, uses strategies to evade the host’s adaptive immune response. One of these strategies is the expression of antigenically distinct VSG on the plasmatic membrane. Cross et al. in an analysis carried out in 2014, obtained, for the Lister 427 strain of *T. brucei*, an assembly of over 2500 genes coding for VSGs; about 80% comprise pseudogenes and partial genes [62]. Despite the vast number of VSG genes in *T. brucei*, only one is expressed at any time in the cell membrane of the parasite. These VSGs are polycistronically transcribed by pol RNA 1 from specialized units known as bloodstream expression sites (BES). Numerous studies have revealed that the BES containing the expressed gene have a lower concentration of nucleosomes, indicating a decompacted and active state in that area of the genome [63,64,65].

It has been reported that various chromatin regulators are essential for the proper silencing of BES, such as CAF-1b, a replication-dependent histone chaperone, and the replication-independent chaperone ASF1A. The knockdown of the ASF1A and CAF-1b genes caused the suppression of repression in the different stages of the parasite’s cell cycle, causing changes in the transcription states of the different VSGs. Therefore, these chromatin regulators act as modulators of the cell cycle and the differential expression of VSGs [66]. On the other hand, the chromatin remodeler *Tb*ISWI, the histone deacetylase DAC3, and the depletion of nucleoplasmin-like protein (NLP), a transcription regulator, increased the expression levels of silenced BES. However, the exact role of NLP in modulating BES is not known with certainty [66,67].

In contrast, the only factor associated with the activation of BES is the high mobility group protein TDP1 [68]. In addition, it has been reported that the histone methyltransferase DOT1B trimethylates the lysine 76 of histone H3, which is involved in VSG switching [69,70]. Later, Aresta et al. in 2016, documented that TDP1 is an essential part of the BES being actively transcribed. TDP1 is an architectural chromatin protein that is an essential high mobility group box (HMGB) that is particularly abundant in the chromatin of active BES and at rRNA genes. In addition, it is necessary for their transcription [65,68]. Therefore, a TDP1 inhibitor could diminish the transcription of any BES, compromising the parasite’s viability. Furthermore, there was a relocation of genes transcribed by pol RNA 1, among them the VSG, which was transcriptionally active when associated with the periphery of the nucleolus. On the other hand, the positioning at the periphery of the nuclear envelope is accompanied by gene silencing by chromatin condensation [71].

### 3.2. Histone Post-Translational Modifications (HPTMs)

Regulating gene expression through HPTMs has been considered a potential therapeutic target for treating sleeping sickness. Four orthologue (*Tb*DAC1–*Tb*DAC4) proteins have been reported for histone deacetylases in *T. brucei*, as well as three homologous sirtuin proteins including *Tb*SIR2rp1 located in the nucleus and TbSIR2rp2 and TbSIR2rp3 in the mitochondria [72,73]. All four *Tb*DACs, except for *Tb*DAC2, preserve the critical residues for acetylation/deacetylation. Through gene replacement strategy experiments based on homologous recombination, Ingram et al. showed that *Tb*DAC1 and *Tb*DAC3 are essential for parasite viability. Additionally, *Tb*DAC4 seems to be involved in a delay in initiating the M phase. The authors conclude that *Tb*DAC1 and *Tb*DAC3 are potential therapeutic targets because RNA interference (RNAi) approaches showed that the knockdown of both genes was detrimental to maintaining parasite survival. Finally, with the help of recombinant expression experiments, it has been demonstrated that *Tb*DAC1 and *Tb*DAC3 play key roles in VSG gene silencing in the different parasite stages [74,75,76].

*T. brucei* has histone-modifying enzymes such as *Tb*PRMT7, a protein arginine methyltransferase (PRMT) responsible for monomethylating Histone H4, which seems essential for the establishment and maintenance of a wide range of chromatin modifications associated with transcriptional activation; thus, the inhibition of this kind of PRMT could be detrimental to parasite survival [77,78].

**Figure 5:**
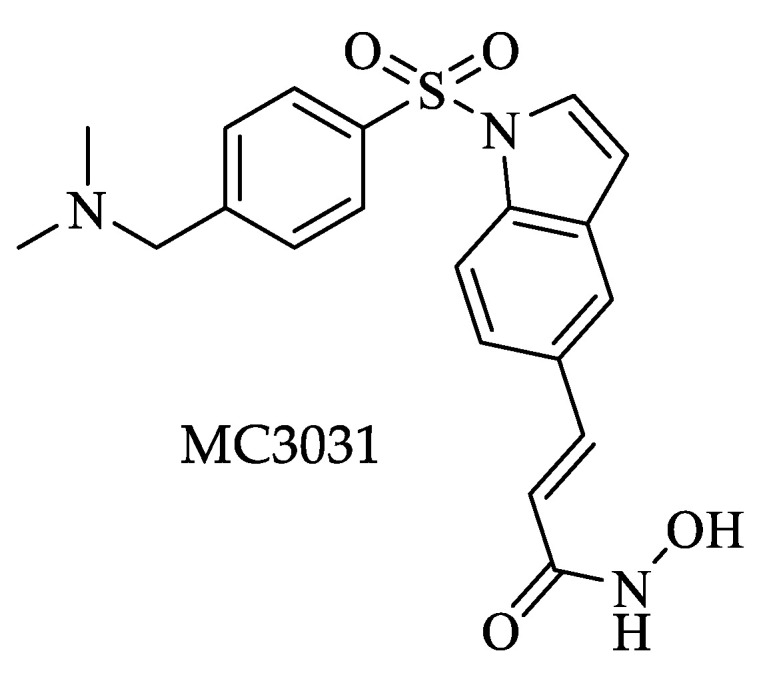
Structure of compound MC3031 with epigenetic effects against *T. brucei*.

Furthermore, Wang et al. screened a variety of hydroxamate/benzamide-based compounds (23 small molecules) with the highest inhibitory activity against human lysine deacetylase (KDAC) on a representative group of parasites of medical importance, including *T. brucei*, to determine their possibility as potential drugs [79]. Using the Markov algorithm, the authors predicted the existence of proteins belonging to the KDAC family within the proteome of each parasite. Next, in silico drug screening analyses were performed against the different parasite proteins and control groups of mammalian cells, delimiting those with better parasite versus host-cell selectivity. Subsequently, those KDAC isotypes of parasites inhibited in the screening had their structures modeled by homology using crystallized human KDAC as a template. Finally, molecular docking analyses were performed, and the best poses for each selected compound were obtained. The best compound against *Trypanosoma* was MC3031 (Figure 5), with an IC_50_ of 0.267 nM [79]. The results showed that the KDAC1 of most parasites presents a high degree of conservation, except for kinetoplastids parasites, including *T. brucei*, which had the lowest identity percentage (~40%), highlighting that the amino acids on the active site were poorly conserved. The authors reported the existence of three additional possible active sites exclusive to this group, attributable to the high degree of divergence of kinetoplastids. These results suggest that the trypanosome KDAC1 can be susceptible to inhibitory drugs of this enzyme that would not affect the corresponding host KDAC1 [79].

### 3.3. Histones Variants

Regarding histone variants, a study of the complete genome of *T. brucei* revealed the existence of four histone variants: H2A.Z, H2B.V, H3.V, and H4.V. The first two are associated with transcriptionally active sites. The rest are associated with termination sites. Additionally, poorly conserved promoter regions have been reported in trypanosomatid genes, including a very diffuse TATA box; therefore, it has been speculated that the transcription start, and termination sites are delimited mainly by histone variants and HPTMs. These two elements would cause regional changes in the chromatin; in turn, these changes would serve as benchmarks for recruiting numerous proteins associated with chromatin [80].

### 3.4. RNA Interference

As in higher eukaryotes, the RNAi pathway has been shown to play an important role in the biology of different protists. *T. brucei* was the first parasite where this epigenetic mechanism of controlling gene expression was demonstrated [81,82]. Transfection with dsRNA of α-tubulin mRNA led to specific degradation of its homologous cellular RNA, which caused a decrease in this protein in the cell, ultimately preventing cytokinesis and triggering parasite death [81,82]. The α-tubulin deficiency induced changes in parasite morphology, multinucleated cells, and defects in the flagellar axoneme [81]. This RNAi method using dsRNA for degradation of its homologous mRNAs is not exclusive to α-tubulin. It could be exploited as a therapeutic resource.

Additionally, a protein of the Argonaute family in *T. brucei* (*Tb*AGO1) has been reported as one of the few early divergent organisms where the RNAi pathway has been described. The function of this protein would not only be its participation in the RNAi machinery, but it would also stabilize the genome and be responsible for silencing retroposons, according to *Tb*Ago1 gene knockout experiments [83].

In addition, *Tb*DCL1 and *Tb*DCL2 are two Dicer-like proteins involved in the RNAi pathway; *Tb*DCL1 is located in the cytoplasm, while *Tb*DCL2 is in the nucleus. *Tb*DCL1 shows a low level of conservation in relation to the Dicer of higher eukaryotes. According to Patrick et al., *Tb*DCL1 could be the enzyme responsible for siRNA biogenesis from retroposon elements that may be detrimental to the parasite. At the same time, *Tb*DCL2 could be responsible for siRNA biogenesis from chromosomal internal repeat transcripts that accumulate in the nucleolus [84]. The existence of enzymes related to the RNAi pathway is proof of the existence of epigenetic mechanisms in these early divergence organisms, which could be exploited for treating the diseases they produce.

From the plethora of scientific articles on RNAi in *T. brucei*, it is worth highlighting the RNAi analysis carried out on chromosome 1 and its effects on total gene expression performed by Subramaniam et al. in 2006. The early divergence of the kinetoplastid group means that more than half of the open reading frames (ORFs) in *T. brucei* do not have homologs in higher eukaryotes, precluding bioinformatic analyses from predicting possible gene functions. The results of these authors showed that nearly 12% of genes that were knockdowns of 369 predicted ORFs for chromosome 1 were lethal for the parasite. In addition, just over 12% of knocked-down genes resulted in severe defects in parasite growth; finally, ORFs related to cell cycle progression were also annotated [85]. Although this type of study has not been extended to the rest of the chromosomes, it can be extrapolated that there is a considerable number of ORFs that, if knocked down, would compromise the viability of the parasite, and could be exploited for the research of treatments against African trypanosomiasis. Finally, a comparative analysis of the three main species of trypanosomatids of medical importance, *T. brucei*, *T. cruzi*, and *L. donovani*, revealed a group of widely conserved proteins between these species without an apparent homolog in the rest of the eukaryotes. The genes from which this group of proteins derives form a syntenic block in the three species, representing more than 75% of common genes. Therefore, these gene expression silencing methods could be extrapolated to these other species, which lack the RNAi pathway [86].

### 3.5. Base J

The DNA of kinetoplastid flagellates has a highly modified nitrogenous base known as beta-D-glucopyranosyloxymethyluracil or base J. This modification, apparently exclusive to this group, is abundantly represented in telomeric repeats of these organisms. Although the distribution of base J in some kinetoplastids is almost restricted to telomeres, in *T. brucei*, there are other regions with a high base content, for example, in inactive expression sites of VSG but not in the VSG being expressed. Such a characteristic indicates that base J plays a role in regulating transcription. In addition, there is a total absence of base J in the life cycle parasitizing the insect [87,88,89,90,91].

Much has been speculated about the possible function that base J may have, including that it has a function similar to methylated cytokines in higher eukaryotes. For example, it could play an important role in gene silencing, especially for those VSGs that are not expressed, or impede homologous recombination. Furthermore, it has been speculated base J has a role in stabilizing the long series of repeats found in the whole genome of this parasite [90,91,92]. The enzymes of the base J synthesis pathway could be a therapeutic target compromising the expression of certain vital genes for the parasite, considering that they do not exist in mammals.

## 4. *Leishmania* spp.

About 30 protozoa species of the genus *Leishmania* of the Trypanosomatidae family are currently registered; of these, only 20 can cause leishmaniasis in humans. There are variations in the disease caused by the different *Leishmania* species [93,94,95,96,97]. Leishmaniasis is usually classified into three main forms: visceral, also known as kala-azar, the most lethal and with a high mortality rate if not treated properly [97]; cutaneous, which is the most prevalent form; and mucocutaneous [95,96,97,98,99].

According to the WHO (2022), the group of diseases known as leishmaniasis represents a great burden for the poorest populations on the planet. These infections are often complicated by other factors such as malnutrition, population displacement, poor housing conditions, compromised immune systems, and a lack of economic resources [97,100].

**Figure 6:**
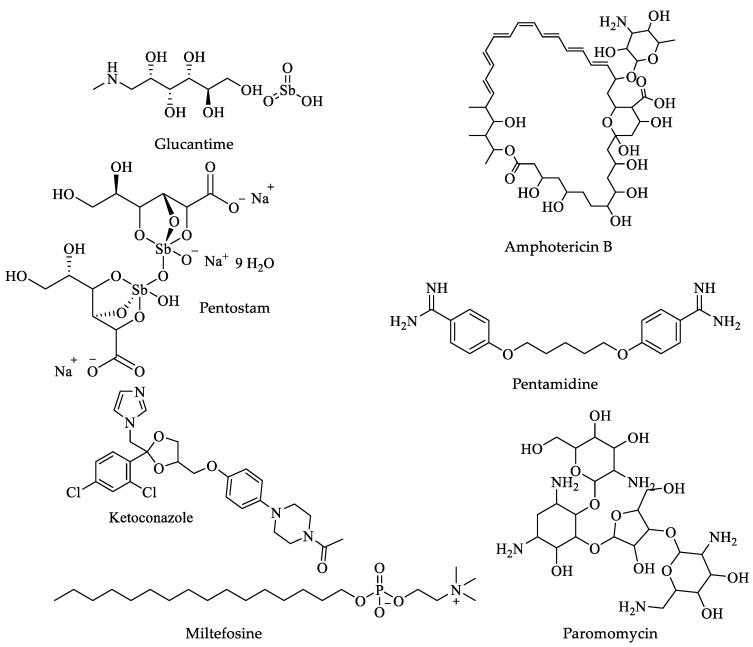
Drugs used for the pharmacological treatment of Leishmaniasis.

The first-line treatment for the different clinical forms of the disease has been pentavalent antimony salts, such as meglumine antimoniate or patent drugs (Glucantime^®^, Aventis) and sodium stibogluconate (Pentostam^®^, GSK). Additionally, other drugs, such as amphotericin B and pentamidine (used in visceral and cutaneous leishmaniasis), ketoconazole and miltefosine (second-line drugs in the treatment of visceral leishmaniasis), and paromomycin (Figure 6) have been tested. Table 3 shows the biological activity, dose, and adverse effects of these leishmanicidal drugs [97,100,101,102,103,104,105,106,107,108,109,110,111,112].

Among the different epigenetic mechanisms described in eukaryotes, only those corresponding to HPTMs and the non-coding RNAs discussed below are well documented in *Leishmania* spp.

### 4.1. Histone Post-Translational Modifications (HPTMs)

Although HPTMs have been extensively studied in eukaryotes, information about them is lacking in early-branching eukaryotes such as trypanosomatids. For example, histone sequences between trypanosomatids and the other eukaryotes are not as well conserved. A lack of promoter regions and terminator elements in kinetoplastids genomes has been reported. It is hypothesized that canonical histone variants and the presence of the DNA base J play important roles in supplying both promoters, and terminators. The histone variants H2A.Z and H2B.V have been reported as crucial for the survival of *L. major* [113].

Additionally, the first genome-wide maps of DNA-binding protein occupancy in *Leishmania major* (*L. major*) suggested high levels of acetylated H3 histone in the start regions of the polycistronic units of protein-coding genes. Therefore, a general transcriptional regulation can be carried out by modifying the acetylation levels of these polycistronic transcription units [114].

One of the main HPTMs is the acetylation carried out by histone acetyltransferases (HAT). A particular case is HAT2 in *Leishmania donovani* (*L. donovani*), which has been reported as essential for proper growth and cell cycle progression, with a low survival rate of parasites lacking such an enzyme necessary for histone acetylation (H4K10). The latter HPTM modulates the transcription of CYC4 and CYC9 cyclin genes for proper cell cycle progression [115].

According to Chandra and coworkers (2017), *L. donovani* has two levels of transcription regulation. It has been reported that *Leishmania* has gene groups that are transcribed as a single polycistronic mRNA. This polycistronic transcription unit is the subject of the first level of regulation; some genes, such as those for cyclins, have promoters, constituting the second level of transcriptional modulation. Therefore, parasites with the HAT2 gene knockout would have a more sensitive effect on H4K10 acetylation levels in the promoter region of the CYC4 and CYC9 cyclin genes [115].

It has been reported that knocking out the HAT3 gene in *L. donovani* led to failures in histone deposition and negatively impacted parasite viability, suggesting its role in modulating cell growth and proper cell cycle progression [116]. It was also reported that HAT3 was associated with the protein proliferating cell nuclear antigen (PCNA), which is necessary for DNA repair after UV exposure [116]. Therefore, HAT2 and HAT3 can be therapeutic targets for drug design.

In other *L. major* studies, it was shown by Southern blot assays that rRNA genes are associated with a histone core. Additionally, through chromatin immunoprecipitation (ChIP) assays, several acetylated histones, such as H3K14ac, H3K23ac, and H3K27ac, were enriched in the promoter region of rRNA genes. These studies also reported the existence of characteristic HPTMs of heterochromatin, such as H4K20me3 (trimethylation), which were present in intergenic spacers and some coding regions but absent from promoter regions of rRNA genes [117].

Other histone-modifying enzymes, perhaps the most studied for their inhibition, are histone deacetylases (HDACs). The presence of four genes whose products are HDACs and three whose products are proteins homologous to sirtuins in higher eukaryotes has been reported in *Leishmania* spp. [118]. Given the divergence between parasitic HDACs and their orthologs in humans, they are promising therapeutic targets for treating parasite diseases. A case to be highlighted is lysine deacetylases (KDAC), which have high divergence in *Leishmania* compared with the host, making them an ideal therapeutic target [119,120]. A first approximation was carried out by Loeuillet in 2018, who tested a variety of compounds, including aminophenylhydroxamate, and aminobenzylhydroxamate derivatives. The authors reported HDAC inhibitor values of IC_50_ > 15 μM in the best cases but with high cytotoxicity in different human cell lines. Additionally, these compounds had good HDAC inhibitor values against *Toxoplasma gondii* (*T. gondii*), as discussed in the corresponding section [121].

Furthermore, Melesina et al. compared the available X-ray structures of human and parasite HDACs. Additionally, the authors modeled the three-dimensional structure of 26 HDACs from 12 parasites by homology. It is worth mentioning that all the modeled structures had at least 40% similarity compared with their human HDAC ortholog. The authors found that the binding pocket region was highly conserved in all the analyzed enzymes. The region on its western side was very similar in all resolved crystallographic structures and the structures predicted by homology. This western side consists of glycine and two phenylalanine residues. Another conserved region includes the residues coordinating the zinc atom (D176, H178, and D264 regarding *Hs*HDAC1) [122]. In contrast, the least conserved region was located on the eastern side of this pocket, which presents, depending on the species, the formation of sub-pockets that can be used to design a variety of exclusive *Leishmania* inhibitors [122].

**Figure 7:**
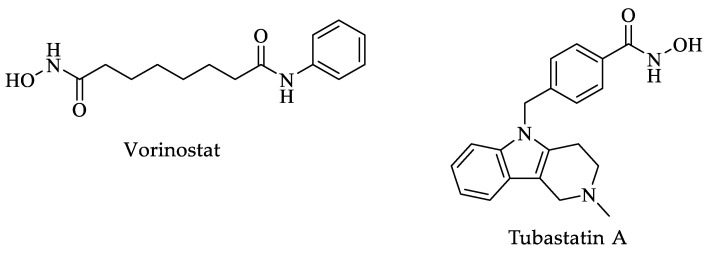
Pan-HDAC inhibitors as antileishmanial agents.

According to molecular docking analyses, various compounds with possible inhibitory activity against HDACs were identified. These compounds had different sub-pockets on the eastern side as binding sites. The authors tested a variety of pan-HDAC inhibitors, such as vorinostat (Figure 7), trichostatin A (Figure 2), and tubastatin A (Figure 7), corroborating that the inhibitory effect is variable among the different parasites. Finally, this study suggests using isoform-specific HDAC inhibitors, such as J-shaped human HDAC inhibitors, which are suggested as a starting point because they had IC_50_ values of 0.3 to 0.6 μM. It is worth mentioning that the J-shaped human HDAC inhibitors showed IC_50_ values in the μM range against *T. brucei* and *T. cruzi*.

**Figure 8:**
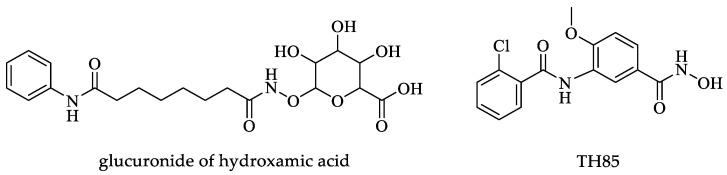
HDAC inhibitors as potent antileishmanial agents with low cytotoxicity.

Ângelo de Souza et al. conducted a study in which 78 compounds derived from hydroxamic acid (Figure 8), a known HDAC inhibitor, were evaluated for their leishmanicidal activity and their possible cytotoxic effect against macrophages. Seven of these compounds showed low cytotoxicity for macrophages, while the inhibitory effect against *Leishmania braziliensis* (*L. braziliensis*) was high. The authors determined the CC_50_ and EC_50_ in macrophages and parasites, respectively. The SI was determined from these assays. Interestingly, two compounds with the greatest efficacy against *Leishmania* intracellular amastigotes obtained SI values greater than 10. Unfortunately, these two compounds were completely innocuous against the promastigote form. Five compounds had SI and EC_50_ values ranging from >3 to 7.6 and 7.21 to 26.5 μM, respectively. TH85 (Figure 8) was the compound with the best activity, with an EC_50_ of 7.21 μM, a CC_50_ of 54.59 μM, and an SI of 7.6 [120]. Furthermore, the authors determined the mode of action of the HDAC inhibitor compounds. The compounds act at the gene expression level and deacetylate other proteins that play crucial roles in cell processes such as apoptosis and cell cycle modulation. Through transmission electron microscopy analyses, they found impaired chromatin compaction and the hallmark of cell processes for apoptosis [120].

**Figure 9:**
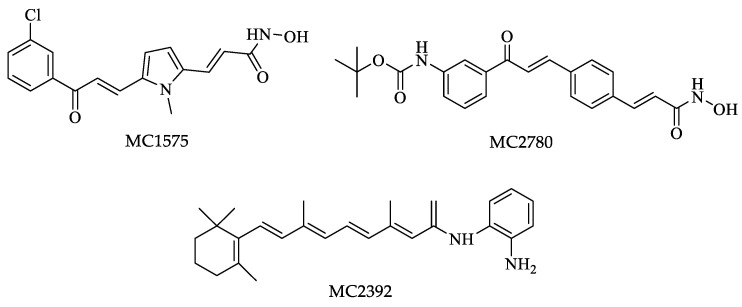
HDAC inhibitors active against *Leishmania* spp.

In a similar study, Di Bello et al. tested a variety of compounds from an in-house chemical library as HDAC inhibitors against *Leishmania* promastigotes. Compounds MC1575 and MC2780 (Figure 9) had a potent HDAC inhibitory activity; however, both compounds also had high cytotoxicity against human cells, while MC2390 (Figure 9) showed low inhibitory activity and no toxicity against mammalian cells. The latter is a starting point for further investigation. Cytotoxicity would be the restricting factor for developing drugs with HDAC inhibitory activity against *Leishmania* due to the high conservation of binding pockets between the HDACs of different eukaryotes [123].

**Figure 10:**
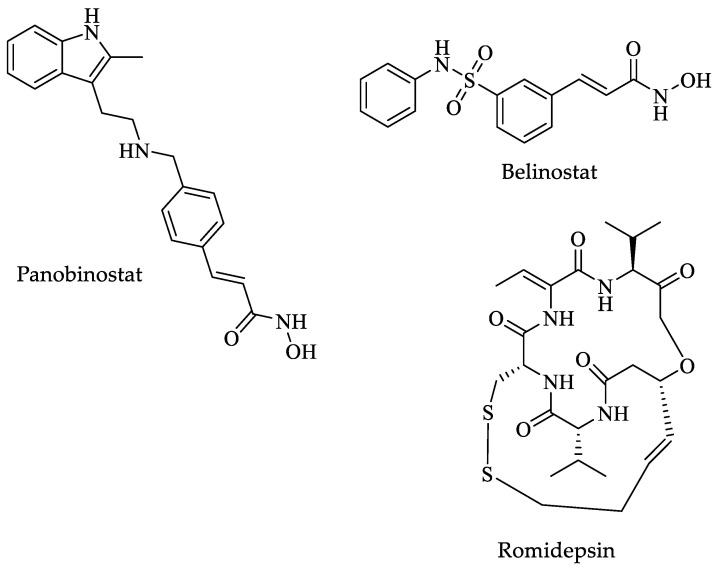
Anticancer drugs evaluated as HDAC inhibitors against four protozoa.

In another study, four clinically approved anti-cancer drugs, vorinostat, belinostat, panobinostat, and romidepsin (Figure 10) with deacetylase activity, were evaluated against *Plasmodium knowlesi* (*P. knowlesi*), *Schistosoma mansoni* (*S. mansoni*), *Leishmania amazonensis* (*L. amazonensis*), and *L. donovani* parasites. None of the drugs had HDAC inhibitory activity against *Leishmania* spp. amastigotes or promastigotes, but they were effective against *P. knowlesi* (IC_50_ from 9 to 370 nM). The compounds displayed a low level of cytotoxicity against other protozoa and flatworms [124].

**Figure 11:**
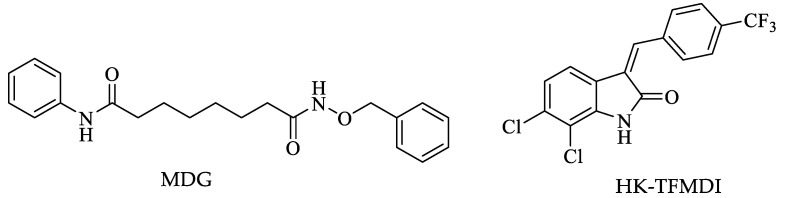
HDAC inhibitor and sirtuin inhibitor active against *Leishmania*.

Finally, in a review, Fioravanti and coworkers analyzed the effects of various HDAC inhibitors. Several of the compounds reported in the literature are not effective as HDAC inhibitors against *Leishmania* parasites, while those with a high inhibitory rate of the enzymes mentioned above were usually cytotoxic in mammalian cells. From the review of these authors, a vorinostat (Figure 7) derivative stands out as the best inhibitory compound against *Leishmania* and had no cytotoxic effects in mammalian cells. This compound, called MDG, was innocuous against promastigotes but exceptionally effective against *Leishmania* strains in the intracellular amastigote form. It is pertinent to mention that this compound, absorbed with gold nanoparticles, decreased the parasite load in vivo. Additionally, only compound HK-TFMDI (Figure 11) had inhibitory potential against the putative sirtuins in this parasite [118].

### 4.2. Non-Coding RNAs (ncRNA)

In eukaryotes, some gene expression is regulated by the interaction of mRNA with non-coding RNAs (ncRNAs). Despite key RISC components reported in *Leishmania*, analysis of the parasite’s genome reveals that it lacks the Drosha enzyme needed for identifying and cleaving double-stranded RNA [125]. Furthermore, the success of parasite adaptation to different hostile environments is attributed to rapid and important changes in gene expression profiles. It is postulated that ncRNA modulates gene expression [126].

In 2019, the first comparative study of the *Leishmania* transcriptome in the different stages of the parasite life cycle revealed a clear difference in gene expression profiles of both protein-coding RNAs and ncRNAs. As in other eukaryotes, putative ncRNAs can function as signaling molecules or regulate gene expression. Their study is crucial for understanding gene expression and regulation processes [126]. Due to their importance, ncRNAs can be used as a therapeutic target to combat the diseases caused by this group of parasites. The presence of ncRNAs regulated through parasite development was reported for the first time in 2006 in *L. infantum* and *L. donovani* at the amastigotes stage. Since the amastigogenesis process is crucial for infection of the mammalian host macrophages, characterization of ncRNAs specifically expressed during the amastigote stage may help us understand the mechanisms of parasite stage-specific gene regulation. This finding was achieved using a genomic library that differentially hybridized against total RNA probes from different life cycle stages (amastigote or promastigote). For *L. infantum*, a new type of noncoding RNA was identified, which varied in size between 300 and 600 nucleotides [127].

Finally, a group of ncRNA characteristics of kinetoplastid protozoa that play an important role in the editing of the genes included in the mitochondrial genome (kDNA) is worth mentioning. The editing process includes inserting and deleting uridine bases at specific sites in the mitochondrial pre-mRNAs. The sequence-specific editing process of mitochondrial pre-mRNAs is carried out by oligo-uridylated small non-coding RNAs, also known as guide RNAs (gRNAs). Additionally, the editing process involves a high molecular weight complex named editosome made up of proteins. This editosome is responsible for forming initiation codons and correcting changes in the reading frame to produce mitochondrial proteins [128,129,130]. Although these gRNAs merit further study, it is tempting to suppose that drug suppression of these gRNAs or inhibiting those proteins that form the editosome, which is exclusive to this group, could impair parasite viability, as it may inhibit the transcription of genes essential for parasite survival such as cytochrome oxidase subunit III (COIII) [131].

## 5. *Entamoeba histolytica*

*E. histolytica* is an intestinal parasite that infects humans and other primates. It is the causal agent of amoebic colitis and amoebic liver abscess and one of the leading causes of diarrhea worldwide [132,133,134]. It is estimated that more than 50 million people are infected by *E. histolytica* each year, in addition to being responsible for the death of almost 100,000 people annually, mainly in developing countries. [135]. *E. histolytica* infection begins by ingesting parasite cysts from fecal-contaminated food or water. Afterward, these tetra-nucleate cysts give rise to trophozoites in the small intestine of the host. They then migrate to the colon, sometimes penetrating the epithelium to spread through the bloodstream [134,136,137,138,139].

**Figure 12:**
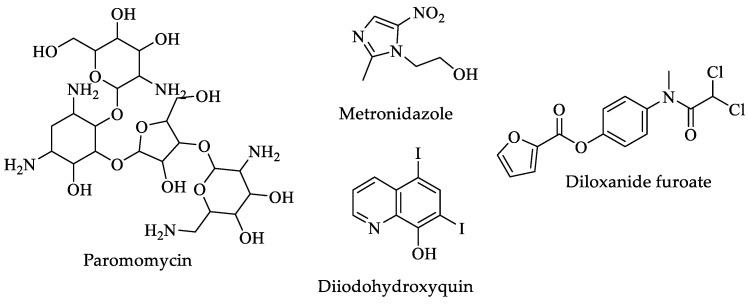
Main drugs used for pharmacological treatment of amoebiasis.

Treatment for this parasitosis depends on the type of infection; for example, noninvasive infection is usually treated with paromomycin (Figure 12), which normally eliminates parasites from the intestinal lumen. On the other hand, an invasive infection is preferentially treated with metronidazole (Figure 12) or any other drug from the nitroimidazole group. Unfortunately, these drugs are only effective against the trophozoite stage. Diiodohydroxyquin and diloxanide furoate (Figure 12) are effective against *Entamoeba* strains resistant to first-line drugs [136,137,138,140]. They are usually prescribed as second-line drugs. Their biological activity, dose, and adverse effects are shown in Table 4.

Numerous studies have discovered that *Entamoeba* virulence can be altered by the environment and interactions with other microorganisms [131,132]. These studies suggest the possible existence of epigenetic regulatory mechanisms that could play an important role in the biology of this parasite. Therefore, this section lists epigenetic mechanisms that can be therapeutic targets against this parasite.

### 5.1. Histone Post-Translational Modifications (HPTMs)

An analysis of the *E. histolytica* genome identifies genes coding for histone-modifying enzymes and various HPTMs. This finding suggests the existence of a gene expression regulation pathway, which could be used as a therapeutic target to combat this medically important parasite [143,144,145,146].

Although the amino acid sequences of histones H3 and H4 in *E. histolytica* have been reported as divergent from those in higher eukaryotes, the amino acid residues involved in HPTM activity are well conserved [144,145,146]. These HPTMs play important roles in regulating different biological processes of this parasite; an example is encystment. Since the encystment process cannot be induced in *E. histolytica*, *E. invadens* served as a human parasite model. A combination of histone acetylation and deacetylation modulates the expression profile of certain genes involved in chitin synthesis and cyst wall formation [147,148].

Studies have demonstrated that during the life cycle of *E. invadens*, the amino-terminal region of the H4 histone was differently acetylated in various Lys residues, particularly at 5-, 8-, 12-, and 16-positions. Subsequently, using the RNA-seq technique, the changes in the *Entamoeba* transcriptome in response to trichostatin A (Figure 2) were analyzed. Significant changes in the levels of H4 acetylation were found, especially in those that code for enzymes involved in synthesizing chitin and decreasing those of the glycolysis pathway in the differentiation of trophozoite to cyst [147,148]. Because the cyst is the infective form and is essential for survival outside the host, inhibiting enzymes that regulate the encystment process could be a valuable tool for treating amoebiasis.

In another study related to HDAC inhibitors, it was shown that two inhibitors, a short-chain fatty acid (SCFA) and trichostatin A (Figure 2), downregulated genes involved in parasite virulence, such as cysteine proteases, lysozyme, and the virulence factor Gal/GalNAc lectin [149]. By comparing whole genome expression profiles, a detailed response to both HDAC inhibitors was observed: while both inhibitors increased histone H3 and H4 acetylation, only trichostatin A (Figure 2) was associated with significant changes in gene expression. It is emphasized in this study that the gene expression profile discovered in *Entamoeba* trophozoites treated with this inhibitor significantly overlaps with the changes in gene expression that are indicative of the trophozoite’s transformation into a cyst [149]. Therefore, the use of other inhibitors of histone-modifying enzymes should be investigated.

Other histone-modifying enzymes, such as histone methyltransferases, catalyze the transfer of methyl groups to arginine or lysine residues on histones H3 and H4. Histone methylation is an epigenetic mark that can activate or repress gene expression. The alteration of gene expression is primarily influenced by the location and number of methylations present in the amino acid residues [150,151].

Arginine methylation is catalyzed by enzymes known as protein arginine methyltransferases (PRMTs). Five putative PRMTs in the *Entamoeba* genome (*Eh*PRMTs) have been identified by in silico analysis [136]. Subsequently, it was found that three of the PRMTs present structural homology to the PRMT1 of *Homo sapiens*. They are expressed in the nucleus of the trophozoites and exhibit histone H4 methylation activity [151].

Lysine methylation is catalyzed by lysine methyltransferases (HKMT). In an in silico analysis in the *Entamoeba* genome searching for genes encoding proteins with the SET domain characteristic of these enzymes, four putative *Eh*HKMTs were identified. Experimental evidence indicated that the four proteins could methylate lysines on histones H3 and H4; furthermore, these enzymes had a primarily nuclear localization, although they also colocalized in the cytoplasm of trophozoites [152]. Since methylation of specific lysine residues has been reported in other eukaryotes to act as a hallmark to recruiting chromatin remodeling complexes [153], it was hypothesized that they might play the same role in *Entamoeba* as a mechanism for regulating gene expression. Evidence for this has previously been reported, where the H3K27Me2 mark was associated with heterochromatin. Additionally, the demethylation of H3K4 on the *Eh*ap-a gene is a sign of gene silencing that inhibits the synthesis of the amoebapore virulence factor [152,154].

**Figure 13:**
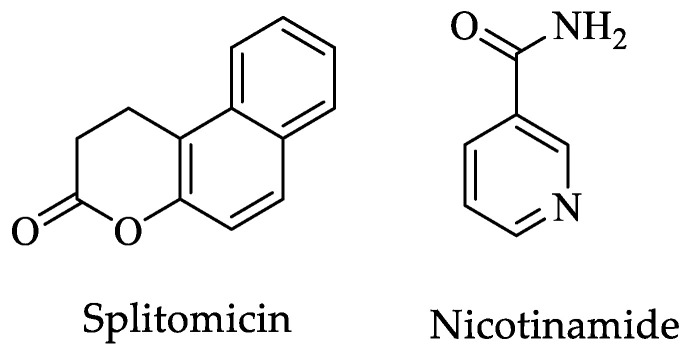
Sir2 inhibitors as antiamoebic agents.

In addition, Dam et al. reported that the *Entamoeba* genome has four genes coding for sirtuins, which are protein deacetylases. *Eh*Sir2a was found in the cytoplasm and nucleus according to immunolocalization analysis. Furthermore, using yeast two-hybrid library screens it was found that *Eh*Sir2a interacts with at least seven proteins, including α-tubulin, which would act as a deacetylase, destabilizing microtubules [155]. Therefore, *Eh*Sir2a would have a role in regulating the function of the cytoskeleton and modulating the transcription and translation of proteins; a function in the synthesis and degradation of lipids was also suggested. Finally, the authors reported the impact of three Sir2 inhibitors, including sirtinol (Figure 2), splitomicin, and nicotinamide (Figure 13), on the *Entamoeba* homologous enzyme. They discovered that splitomicin did not inhibit *Eh*Sir2a, but sirtinol and nicotinamide had IC_50_ values of 12.47 µM and 0.79 mM, respectively. The authors attribute the resistance to this first drug to the lack of a histidine residue, which has previously been reported as crucial for recognizing this compound. According to the authors, an important epigenetic mechanism mediated by *Eh*Sir2a was identified, which regulates the microtubule polymerization of *E. histolytica* [155].

There are a variety of biological processes that seem to be regulated by different epigenetic mechanisms, including HPTMs, so the existence of both methylation marks in *Entamoeba* histones, as well as the existence of PRMT enzymes, is an indication of the important role they play in the regulation of gene expression. Therefore, drugs that inhibit these enzymes should be investigated for the treatment of this disease. From these data, it can be hypothesized that histone-modifying enzymes could be a promising therapeutic target to prevent the transmission of amebiasis caused by *Entamoeba*. Furthermore, protein acetylation by sirtuins is another important mechanism of protein activity regulation [150,151,152,153,154].

### 5.2. RNA Interference

**Figure 14:**
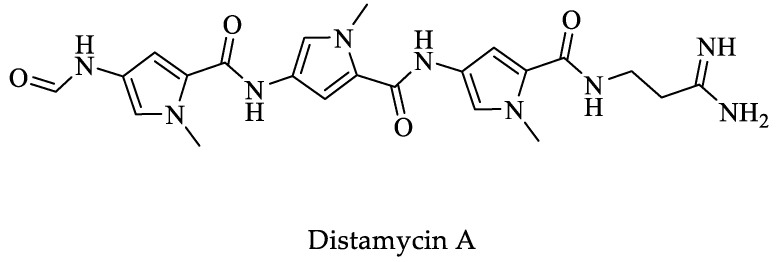
Structure of compound that affects *Eh*MLBP.

The RNAi pathway in *E. histolytica* has been used to silence important genes responsible for virulence in certain strains [156]. An example is reported by Lavi et al. who identified a protein called *E. histolytica*-methylated LINE binding protein (*Eh*MLBP), which specifically binds to the rDNA episome (DNA methylated), and long interspersed nuclear elements (LINEs). It is suggested that *Eh*MLBP performs the function of measuring the levels of repetitive methylated DNA. Downregulation of *Eh*MLBP using synthetic antisense oligonucleotides caused alterations in the growth and pathogenicity of the parasite [157]. Furthermore, treating trophozoites with the drug distamycin A (Figure 14) inhibited parasite growth and pathogenicity because it binds to DNA and interrupts *Eh*MLBP activity to check cytosine methylation levels (m5C). *Eh*MLBP is exclusive to *Entamoeba* without homologs in mammals. According to the authors, *Eh*MLBP is essential for *E. histolytica*. It is a potential target for antiamoebic chemotherapy [157].

In another study, Ankri et al. using antisense technology, inhibited the expression of the light subunit (35 kDa) of the Gal/GalNac lectin complex, which is involved in the adhesion of the parasite to target cells. The decreased protein expression did not affect the adhesion of trophozoites to bacterial or mammalian cells but rather decreased their cytotoxic activity and ability to induce liver abscesses in hamsters. For this reason, the Gal/GalNac lectin complex subunit could play a role in parasite virulence and be considered a potential therapeutic target [157,158].

Mirelman et al. silenced transcription of a gene encoding amoebopores by transfecting trophozoites with a plasmid containing a segment of the 5’ upstream region of the same gene whose silencing continued even after plasmid extraction. These clones were named G3 and were subsequently transfected with a plasmid containing the cysteine protease gene (*Eh*CP-5) and the Gal lectin light subunit gene (*Eh*lgl1) downstream to the 5′ sequence of the amoebopore gene, inducing simultaneous silencing of both genes. Silencing the three genes produced trophozoites (RB-9) with attenuated virulence. Notwithstanding, this new attenuated strain RB-9 expresses the same surface antigens that are expressed in virulent strains, which are sufficient to induce an immune response in hamsters, suggesting the possibility of its use as a vaccine [159].

Finally, Nurkanto et al. analyzed the Coenzyme A (CoA) biosynthetic pathway of *E. histolytica*. They highlighted that pantothenate kinase (PanK) and dephospho-CoA kinase (DPCK) were divergent enough from the human orthologue. Therefore, they were used as targets for gene silencing of their respective genes with a plasmid containing a portion of their corresponding 5′ upstream region. The results indicated that parasite viability was significantly compromised [160,161,162].

### 5.3. DNA Methylation

DNA methylation is a process for silencing gene expression. Searches of the genome of *Entamoeba* indicated that, unlike mammals, this parasite has only one enzyme, *Eh*meth, with (cytosine-5)-methyltransferase activity. The protein showed homology and high structural similarity with the human methyltransferase Dnmt2 and can methylate DNA and tRNA (C38 of tRNA^Asp^). DNA methylation would play an important role in silencing transposable elements, providing stability to the amoeba genome [163,164,165,166].

**Figure 15:**
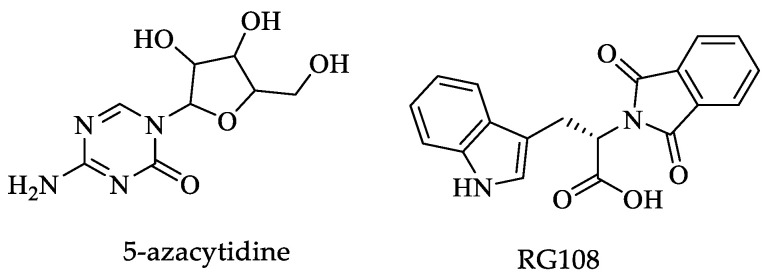
Structure of DNA methyltransferase inhibitors of *E. histolytica*.

On the other hand, it has been reported in *Entamoeba* that the gene coding the heat shock response protein 100 (Hsp100) is methylated in its promoter region. After exposure to the drug 5-azacytidine (Figure 15), an inhibitor of DNA methyltransferases, the HSP100 in the parasites was expressed at levels similar to parasites subjected to heat shock. Furthermore, trophozoites incubated with a concentration of 23 μM of 5-azacytidine decreased their ability to kill mammalian cells and produce liver abscesses in hamsters; however, a high dose of 100 μM was lethal to the parasite but also to mammalian cells. Therefore, the authors suggest using novel non-nucleoside Dnmt inhibitors, such as RG108 (Figure 15) [167].

## 6. *Giardia lamblia*

This parasite causes human giardiasis, one of the most frequent gastrointestinal parasitosis worldwide [168,169,170]. According to the WHO, *G. lamblia* (syn. intestinalis, or duodenalis) is one of the most common agents of diarrheal diseases worldwide, with more than 300 million cases reported annually [171,172]. The incidence of giardiasis in humans is associated with the degree of development of a country, ranging between 2% and 3% in industrialized countries and up to 30% in low-income and developing countries [173,174,175]. *Giardia* eradication from the human population and water sources represents a challenge for researchers and public health authorities [168,169,170,171].

**Figure 16:**
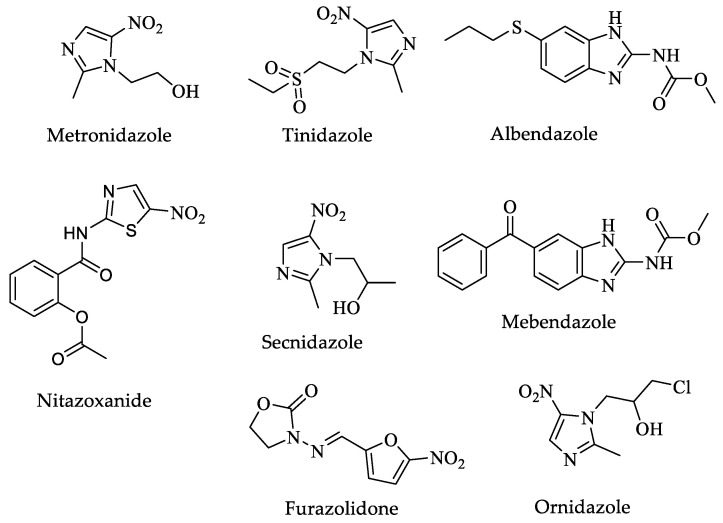
Main drugs used for the pharmacological treatment of giardiasis.

Several drugs are used to treat this parasitosis, in particular, metronidazole, tinidazole, and nitazoxanide (Figure 16) are used as first-line drugs. Albendazole, mebendazole, furazolidone, secnidazole, and ornidazole (Figure 16) are other drugs for treating strains resistant to the first-line drugs. Regardless of the drug used, clinically resistant strains have been reported for several of these drugs. In addition, some have well-documented severe side effects, such as neurotoxicity, optic neuropathy, peripheral neuropathy, and encephalopathy for metronidazole, and genotoxic effects in animal models, with this latter effect being controversial in humans (Table 5) [176,177,178].

*Giardia* trophozoites present antigenic variation to survive within the host small intestine. This is a process by which the parasite continuously switches its major surface molecules, allowing it to evade the host’s immune response and produce chronic and recurrent infections [179].

### 6.1. Variant-Specific Surface Protein

*Giardia* has approximately 190 genes encoding variant-specific surface proteins (VSP). Despite this great variety, the parasite expresses only one VSP on the cell surface at a particular time. In 2006, Kulakova et al. transfected trophozoites to express the VSP7 and, by immunodetection, identified those organisms that expressed the protein. Due to the high similarity between the nearly 200 types of VSPs and their flanking sequences, an epitope with hemagglutinin (HA) was inserted into this vsph7. Despite vsph7 and vsph7-HA being identical genes with identical UTR regions, they were differentially expressed, demonstrating that the mechanism involved in antigenic expression does not depend on the sequence. In the same work, by chromatin immunoprecipitation (ChIP), PCR assays, and specific antibodies against acetylated lysines, these researchers demonstrated that the transcription of vsph7-HA correlates with acetylated lysines on the nearby upstream histones [180].

On the other hand, Prucca et al. hypothesized that the expression of the VSPs could be controlled in a post-transcriptional way; hence, all genes coding for VSPs would be transcribed and subsequently silenced except for the VSP on the membrane surface of the trophozoite. This mechanism would implicate enzymes such as RNA-dependent RNA polymerase (RdRP), Argonaute protein, and Dicer. By knocking down the RdRP and DICER genes, the authors found trophozoites with more than one VSP on their membrane surfaces, which positively supported their prediction [181]. It was proposed that using these VSPs as antigens to produce a vaccine would allow the host to generate antibodies and reactivate cells against the entire repertoire of VSPs to combat the infection and/or the clinical manifestations of the disease [181].

### 6.2. Histone Post-Translational Modifications (HPTMs)

Salusso et al., through an analysis with the HMMER software in the *Giardia* genome database, found three genes encoding putative histone methyl transferases according to the presence of the SET domain present in all HMTs. Through multiple sequence alignment analysis, a high percentage of similarity between *Gl*HMT1 and the HMTs of various species was found, with the four motifs that compose the catalytic site highly conserved. They made a 3D model of *Gl*HMT1 and compared it with the resolved structure of human ASH1 from the PDB database (3OPE), showing a coincidence at the structural level. Afterward, they found overexpression of the *Gl*HMT1 gene during parasite differentiation [182].

The *Gl*HMT1 protein was monitored at different stages to evaluate its role in the encystment process, finding that it was expressed in trophozoites and the early encysted cell stages. Additionally, the downregulation of *Gl*HMT1 induced the upregulation of encystment-specific genes during the early stages of the encystment process [182]. Therefore, HMT inhibitors could stop the differentiation to cysts in *Giardia*, reducing disease transmission.

**Figure 17:**
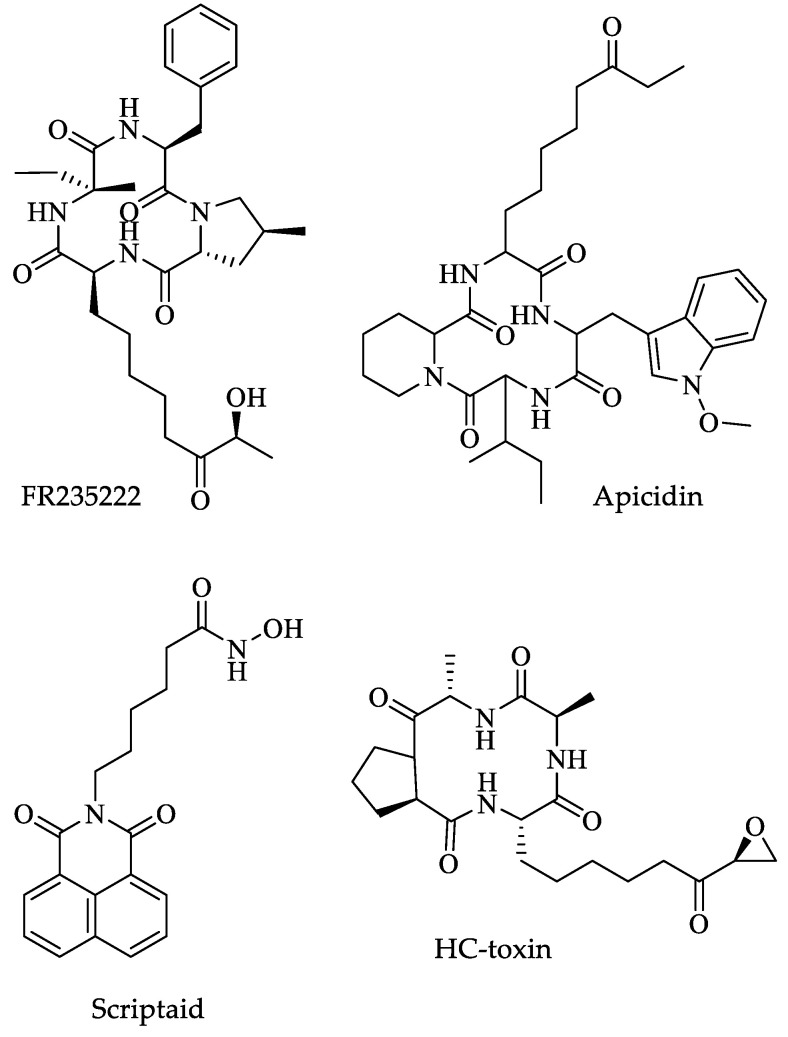
Compounds used to inhibit HDAC against *G. lamblia*.

Another type of HPTM is acetylation at the histone tails, which is responsible for changes in DNA compaction; for this reason, it has been studied for years. Several drugs with anticancer properties have been developed which could have HDAC inhibitory activity [183,184]. Sonda et al. searched the *Giardia* genome database for possible HDAC enzymes finding only one that was homologous to the classical HDAC enzyme and four additional sirtuin-like enzymes. The authors performed molecular docking simulations to determine if the catalytic pocket of the *Giardia* HDAC could accommodate the HDAC inhibitor FR-235222 (Figure 17). They found three binding modes within the HDAC pocket.

Furthermore, another study found that *Giardia* parasites treated with FR-235222 had increased histone acetylation and changed expression pattern in 2% of genes, including those related to encystment [185]. They also found that FR235222 inhibited, at nanomolar concentrations, the expression of the cyst wall protein CWP1, the main component of the cyst wall. The authors also tested other HDAC inhibitors, such as apicidin, trichostatin A, scriptaid, and HC-toxin (Figure 17), which had the same effect in decreasing CWP1 expression. Finally, no negative effect on parasite replication was reported. It is worth mentioning that the same doses were tested in mammalian epithelial cells without affecting viability [185].

Finally, Carranza et al. reviewed the literature for the most common modifications in histones and their relationship with the differentiation process in *G. lamblia*. According to these authors, enzymes such as HDAC and sirtuins regulate the encystment process and antigenic variation. They concluded that high rates of lysine acetylation on histone are essential for encystment and parasite propagation; hence, HDAC inhibitors could impair encystment. On the other hand, low rates of H4K8 acetylation and H3K9 methylation are found during antigenic variation. Additionally, H3K9me3, a repressive epigenetic mark, increased during encystment [186].

### 6.3. RNA Interference

A study by Saraiya et al. reported the existence of a micro-RNA (miR3) derived from a snoRNA located in the nucleolus of *Giardia*. miR3 (26 nt in length) can repress translation of the mRNA encoding histone H2A; such repression occurs through an imperfect alignment of miR3 and the H2A mRNA with the intervention of the giardia Argonaute protein (*Gl*AGO). If miR3 perfectly aligns with the H2A, mRNA enhances its translation. The transition between repressing and activating H2A mRNA translation depends only on the number of base pairs between miR3 and H2A mRNA; repression is maximum when only 8 nt of miR3 are paired, and when the complementarity is 26 nt, translation is activated to the maximum. Therefore, *Gl*AGO inhibitors and the regulation of pairing between miR3 and H2A mRNA could be a possible therapeutic target [187].

In addition, Jian-You et al. analyzed the whole genome of *G. lamblia*, looking for sRNAs and found two main types: endogenous siRNAs and sRNAs derived from tRNAs. Practically no canonical microRNAs were found. According to their studies, the GlDICER knockdown suggests that both types of sRNAs could play important roles in modulating the Giardia life cycle [188].

The authors sequenced the sRNA transcriptome at both cell cycle stages to identify the sRNAs involved in the giardia trophozoite–cyst–trophozoite differentiation process. They found two new endo siRNAs and five new sRNAs derived from tRNAs. Both groups of functional RNAs were upregulated in the differentiation process of the parasite. Furthermore, the authors reported that the differentiation process was diminished after the knockdown of the DICER gene (*Gl*DICER), a protein responsible for the processing of endo siRNAs [188]. The mechanisms of translation regulation of these small RNAs remain to be elucidated.

## 7. *Toxoplasma gondii*

**Figure 18:**
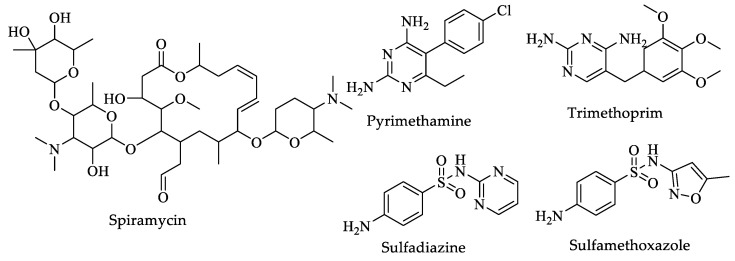
First-line drugs for the pharmacological treatment of toxoplasmosis.

*T. gondii* is a protozoan parasite that belongs to the Apicomplexa group. All members of this group are obligate intracellular parasites of a wide range of homeothermic animals. Apicomplexa parasites present a series of organelles that specialize in the penetration of host cells, known as an apical complex [189,190,191,192,193,194,195,196,197]. According to the WHO, infection by *Toxoplasma* has become a public health problem worldwide [198]. Although the human immune system usually fights acute infection easily, drugs have also been developed to fight this parasite if necessary. The first-line drugs used are spiramycin, a combination of pyrimethamine and sulfadiazine, and trimethoprim and sulfamethoxazole (Figure 18) [199]. Unfortunately, these drugs are highly toxic to the host (Table 6), and there is no treatment for chronic *Toxoplasma* infection. Therefore, it is necessary to identify and design new drugs for treating this infection [193,200].

### Histone Post-Translational Modifications (HPTMs)

*T. gondii* has a complex life cycle, with a great physiological variation between different stages of this parasite; hence, the change from one stage to another requires drastic transcriptional remodeling. A deficiency of general transcription factors was deducted from the genome content analysis of this protozoan. Thus, to achieve changes in the transcription profiles between the different stages of development, *Toxoplasma* has vast chromatin-remodeling machinery [204,205,206].

The first studies to identify HPTMs in *Toxoplasma* were carried out by Saksouk et al. using the ChIP assay. They identified some HPTMs associated with genes involved in the differentiation process between the different parasite stages. They found that acetylation of histones H3 and H4 upstream of constitutively expressed genes increases in tachyzoites and bradyzoites. The bradyzoite stage-specific genes, on the other hand, are hypoacetylated in tachyzoites, altering their levels of acetylation just before differentiation [207]. Next, computer analysis determined the existence of five putative arginine methyltransferases in the Toxoplasma genome. Subsequently, the authors focused their analysis on those enzymes that present an ortholog in higher eukaryotes, such as *Tg*CARM1 and *Tg*PRMT1. The former has as substrate Arg 17 of H3, while the latter Arg 3 of H4. It is worth mentioning that the compound S-adenosylhomocysteine inhibited both methyltransferases with IC_50_ values of 0.04 and 0.40 µM, respectively. Lastly, the authors reported that *Tg*CARM1 inhibition favors the differentiation process, showing that this process has a strong epigenetic component that can be used as a therapeutic target [207].

Even though the *T. gondii* genome has been completely sequenced, understanding how this parasite, and the Apicomplexa group in general, regulate the expression of their genes is still unclear. Analysis of the *Toxoplasma* genome reveals the existence of five putative coding genes for HDAC independent of NAD [208]. These findings suggest that histone modifications, methylation, and acetylation could play important roles in the differentiation stage of this parasite, making them ideal therapeutic targets for treating this disease [204,205,209].

Subsequently, Bougdour et al. demonstrated that FR235222 (EC_50_ 7.6 nM) is a stronger inhibitor than other inhibitors previously tested, such as trichostatin A (EC_50_ 400 nM), pyrimethamine (EC_50_ 285 nM) and apicidin (EC_50_ 15 nM), among others. They showed that FR235222 interacts with toxoplasma HDAC3 (*Tg*HDAC3) by inserting two amino acid residues on the active catalytic site of the enzyme, which consists of the amino acids Ala98 and Thr99. It is worth mentioning that these amino acids are only present on the active catalytic site of the HDAC3 proteins from the Apicomplexa group, being absent from the rest of the HDACs so far identified in other eukaryotes [209]. Hence, this peculiarity could be used for developing inhibitors that only affect the parasite’s enzyme. Furthermore, through ChIP together with DNA microarray (ChIP-on-chip) assays, they identified 369 genes with upstream regions with hyperacetylated nucleosomes after treatment of the parasites with FR235222; interestingly, around one-third of such genes are differentially expressed in distinct stages of the *Toxoplasma* life cycle (sporozoite and bradyzoite) [209].

Another example is reported by Hanquier et al. [210]. They studied the lysine acetyltransferase (KAT) named GCN5. This family of enzymes is represented in the *Toxoplasma* genome by two prospects, GCN5a and GCN5b; each one has a bromodomain at the C-terminal end. This domain is responsible for binding to acetylated lysines. Despite the high similarity between both GCN5s, each seems to have different substrate affinities: *Tg*GCN5-A only targets Lys 18 on H3, while *Tg*GCN5-B targets several lysine residues on H3. Additionally, two *Tg*ADA2 homolog proteins (transcriptional coactivators) have been identified, which interact differently with both *Tg*GCN5 that present ADA2-binding domains [210,211,212]. Contrary to what has been described in other eukaryotes, where it responds to stress and development, the set of genes regulated by *Tg*GCN5 corresponds to surface antigens, micronemes, and peptides involved in host cell binding [211].

**Figure 19:**
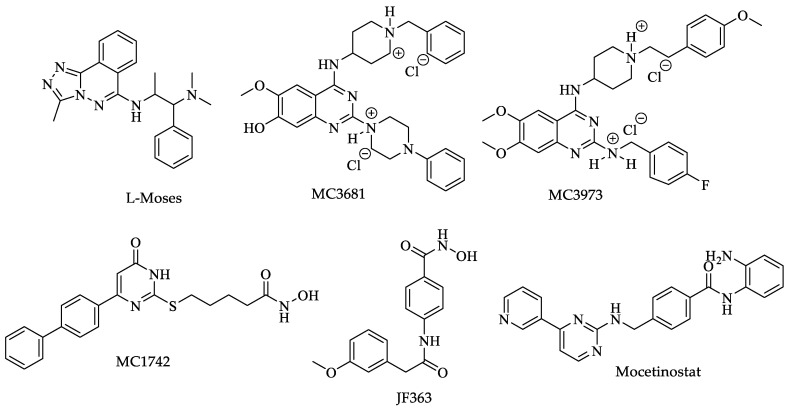
Compounds active against -toxoplasmosis.

Knockout studies on GCN5a indicated that this enzyme is not essential for tachyzoite proliferation but for gene expression in response to alkaline stress. On the other hand, the knockout of GCN5b produces non-viable tachyzoites [210,211,212]. The triazolopthalazine-based chemical compound L-Moses (Figure 19) was tested against this parasite in these experiments. The compound had a high affinity for bromodomains of the PCAF/GCN5 family. The results showed that this drug interferes with the ability of the GCN5b bromodomain to bind to the acetylated lysines of histone tails. The authors found that L-Moses exhibits high GCN5b inhibitory activity on tachyzoites with an IC_50_ of ~0.6 µM. The experiments by Hanquier et al. [210] concluded that the bromodomain of GCN5b is a potential therapeutic target that should be studied.

In addition, Mouveaux et al. tested different compounds with inhibitory activity on histone-modifying enzymes. They identified two compounds, MC1742, and mocetinostat (Figure 19), which had a strong inhibitory activity on HDAC, and two other compounds, MC3681 and MC3973, with an inhibitory effect on DNA methyltransferase (DNMT). All compounds had activity in the micro to nanomolar range [213]. Furthermore, experiments to inhibit DNMT successfully stopped the proliferation of the parasite at concentrations of 10 µM. Unfortunately, this concentration had a significant cytotoxic effect on the cell line used as a control, excluding the possibility of its repositioning [213]. Finally, of the HDAC inhibitor compounds, MC1742 showed the highest inhibitory potential with an IC_50_ value of 30 nM, which was lower than pyrimethamine, the reference drug [213].

The previous examples are not the only ones for drug repositioning against HDAC; other examples are tubastatin A, and vorinostat (Figure 7). These are anticancer drugs that displayed HDAC inhibitory activity against the parasites with IC_50_ values in the nanomolar range and with low cytotoxicity against mammalian cells, making these compounds promising drugs for use against this parasite and possibly others [214]. Additionally, a newly synthesized compound called JF363 (Figure 19) showed in vitro (IC_50_ 0.17 to 0.43 µM) and in vivo (40 or 160 mg/kg) HDAC inhibitory activity [215]. In the same study, the authors performed molecular docking analyses of this compound against the five HDACs found in the *T. gondii* genome (strain ME49); the in silico analysis predicted binding modes on the active site, making JF363 a potential compound that should be further investigated for the treatment of toxoplasmosis [215].

Loeuillet tested aminophenylhydroxamate and aminobenzylhydroxamate derivatives against various parasites. They found a compound named JF363 that was outstanding for its HDAC inhibitory activity against *Toxoplasma*. Its synthesis derives from ST3, a compound with proven anti-leishmania activity. JF363 had HDAC inhibitory values of IC_50_ from 0.35 to 2.25 μM for different *Toxoplasma* strains. The authors report a high selectivity (SI = 300) for the bradyzoite form (intracellular proliferative) regarding human cell lines. It is worth mentioning that there is currently no drug that attacks the intracellular form of the parasite, which remains latent for the entire life of the host, leading to the constant risk of reinfection. The compound presented by Loeuillet showed in vitro IC_50_ values equivalent to the reference drug pyrimethamine, so experiments to test the effect of this compound in vivo should be performed [121].

In summary, the experiments performed to date clearly show that HPTMs, such as histone methylation and acetylation, act as landmarks in promoters of active genes, playing important roles in determining whether a gene is transcribed. Consequently, enzymes that cause epigenetic changes represent potential therapeutic targets and have a promising future as a tool for the fight against toxoplasmosis.

## 8. *Trichomonas vaginalis*

*T. vaginalis* is a flagellated microaerophilic protozoan parasite that adheres to the urogenital tract of humans. It is the etiological agent of trichomoniasis, the largest non-viral sexually transmitted infection in the world and the most curable [216,217,218]. The infection is asymptomatic in almost 50% of women, while over 80% of men have no symptoms. Symptomatic trichomoniasis in women is characterized by vaginal or urethral discharge, pelvic pain, dysuria, and genital itching; trichomoniasis in pregnant women has severe complications such as premature delivery, low birth weight, premature rupture of membranes, and even infertility, whereas men who present symptoms have urethritis [218].

The most used drugs for this infection are metronidazole and tinidazole (Figure 16), although there were cases of resistance to them several years ago (Table 7) [218,219,220,221,222,223,224,225,226,227].

Like other protist parasites, its epigenetic mechanisms are not fully understood. Below are those that may be implicated in regulating vital processes for the parasite and which can be used as therapeutic targets.

### 8.1. Histone Post-Translational Modifications (HPTMs)

Bioinformatics analyses of the *T. vaginalis* genome found a substantial repertoire of histone-modifying enzymes, suggesting that they play an important role in chromatin remodeling and probably in differential gene expression [228]. For example, in a study by Song et al., treatment of the parasites with the HDAC inhibitor compounds apicidin and trichostatin A resulted in significant changes in gene expression profiles, particularly in iron-regulated genes. It is important to point out that research has revealed that iron can control the expression of genes that code enzymes involved in metabolic pathways and virulence factors. Additionally, by RNA-seq, immunoblotting, and ChIP*-*Seq analysis, two histone covalent modifications have been identified, H3K4me3 and H3K27Ac, which are associated with the expression of active genes [229].

Pachano et al. [230] demonstrated the relationship between the degree of histone H3 lysine acetylation (H3Kac) and the active expression of the BAP1 and BAP2 genes by ChIP analysis. These genes encode proteins that adhere to the vaginal epithelium in response to trichostatin A [230].

The authors found that the transcription factor, the initiator-binding protein, requires histone acetylation at the initiator region of most genes to bind to them and initiate the transcription process. This process is necessary for 75% of the protein-coding genes, particularly the BAP1 and BAP2 genes in *T. vaginalis* strains with high adherence capabilities to host cells (strain B7268). In contrast, these genes were lowly expressed and hypoacetylated in the G3 strain, which has less adherence to vaginal epithelium [230]. Afterward, the researchers treated the parasites with trichostatin A, finding that the G3 strain presented an upregulation of these genes and increased H3Kac in regions surrounding the initiator region of both BAP genes [230]. These results showed that the acetylation of H3 lysines is a permissive post-translational modification of key genes that contribute to the adherence of the parasite to the vaginal epithelium and its pathogenicity [230]. Based on this, histone acetyltransferase inhibitors (HATi) could have the opposite effect and be useful for treating this parasitism.

### 8.2. RNA Interference

*T. vaginalis* has been reported to have a larger genome than other parasitic protists; almost two-thirds corresponds to viral DNA, repetitive elements, and transposons. Regarding the RNAi pathway, previous research has identified a supposed RNase III enzyme and two possible Argonaute proteins, which according to phylogenetic analyses, are homologs of PIWI-like AGO proteins from higher eukaryotes [231,232]. The existence of an RNA interference pathway in *Trichomonas* was postulated as part of a defense mechanism to inhibit the activity of transposons [232] that could also play an important role in regulating gene expression.

Warring et al. in 2021 identified a new type of small RNA of ~34 nt by high-throughput RNA sequencing (RNA-Seq) analysis that showed a direct relationship between its expression and decreased gene expression of transposable elements in *Trichomonas*. Due to the similarity between putative *Trichomonas* AGO proteins and the PIWI-like AGO proteins of other eukaryotes, it was suggested that regulation of transposable elements in the parasite would be through piRNA interference (piRNAi) [232]. The authors also identified possible groups of piRNAs within the genome of *Trichomonas* and suggested that the new type of small RNA identified corresponded to piRNAi guides [232]. These results suggest the possible existence of an interference RNA pathway that could play a dominant role in gene expression regulation in *Trichomonas*. This finding supports using AGO and DICER proteins as possible therapeutic targets to control this parasitosis.

### 8.3. Histone Variants

A high number of genes encoding histones that make up the nucleosome of the *T. vaginalis* genome has been reported. This finding suggests the existence of histone variants that could play an important role in regulating gene expression, either by interacting differentially with DNA or by acquiring specific HPTMs. Moreover, a relatively low number of transcription regulatory elements have been found in the genome of this parasite [233,234]. Therefore, the existence of a mechanism for controlling gene expression based on histone variants in this parasite may be possible.

A high number of H3-coding genes has been reported in an analysis performed on the entire *T. vaginalis* genome. Of the putative 23 genes that code for H3, three non-canonical variants have been identified; the rest are identical protein sequences. The three histone H3 variants are called TVAG_185390 (48.9%), TVAG_087830 (95.7%), and TVAG_224460 (49.6%) and have variable percentages of identity concerning canonical trichomonas H3. All were cyto-localized by immunostaining. TVAG_185390 presented a distribution equivalent to the canonical H3. According to multiple sequence alignment, it was found to be sufficiently divergent to affect the nucleosome–DNA interaction or even undergo variant-specific post-translational modifications [228,235].

On the other hand, the variant TVAG_087830 had a greater degree of identity to canonical H3 with a location in transcriptionally permissive sites, and it is enriched in H3K4me marks, the hallmark of transcriptionally active genes [228,235]. Finally, variant TVAG_224460 showed a periphery distribution in the nucleus, associated with the different chromosomes in the interphase stage. It is pertinent to mention that similar studies have not been carried out to search for variants of other histones that are part of the core, considering the diversity of genes that has been reported for the rest of the histones [228,235]. It would be interesting to knock down these genes and evaluate their effects on parasite viability and if they could be used as therapeutic targets for treating this disease.

### 8.4. DNA Methylation

Although the 5-methylcytosine (5mC) DNA modification is relatively well studied in higher eukaryotes, this epigenetic mechanism has not been widely studied in lower eukaryotes [236]. However, in recent years an additional mark on DNA, N6-methyladenine (6 mA), has been reported. It has been found in relatively high levels in the genome of *T. vaginalis*, with a distribution in intergenic regions and certain groups of transposable elements [237]. It has been hypothesized that 6 mA could play some role in regulating the transposition of these elements, which is essential for genome stability. Furthermore, it has been suggested that this covalent modification regulates the expression of genes close to ETs since genes that present this modification are poorly expressed [5,238]. Due to the absence of this mark in the genome of higher eukaryotes, the enzyme responsible for this process (adenine DNA methyltransferases) could be subject to inhibitory drugs that compromise the viability of this parasite.

## 9. Conclusions

Although the diversity of studies on the different epigenetic mechanisms is profusely abundant in recent decades, these studies mainly focus on the molecular and epigenetic processes present in higher eukaryotes, with limited information concerning protists, especially those of early divergence. In some cases, many of these mechanisms as well as the role they may play in the different biological processes of the parasites are still unknown. Given the biology of these parasitic organisms, which involves a diversity of environments and various stages in their life cycles, the role that the different epigenetic mechanisms may have in the different vital processes of the organism has been speculated on for a long time. Therefore, this review addressed the main epigenetic mechanisms described in these parasites.

First, the group of kinetoplastids contains base J, which is particularly associated with telomeres and silencing of LINEs and facilitates the stability of the genome. Additionally, histone variants and HPTMs could play a role in delimiting the promoter regions and terminator elements of the different genes. Therefore, using these mechanisms as therapeutic targets would lead to changes in the modulation of gene transcription, which could be detrimental to the parasite’s viability. It is worth mentioning that HAT and HDAC are highly divergent with respect to their ortholog in humans. Hence, the design of drugs that target these proteins undoubtedly has future therapeutic potential.

Second, the RNAi pathway, reported for the first time in *T. brucei* for the silencing of essential genes, could be extrapolated to both *T. cruzi* and *Leishmania* spp., which share a relatively high number of genes between them, with no homologs in the rest of the eukaryotes. This suggests the RNAi pathway as an important tool for post-transcriptional silencing of genes. Additionally, the mitochondrial pre-mRNA by gRNAs and the editosome itself can be therapeutic targets that compromise the synthesis of key proteins.

In the case of *E. histolytica*, the literature mentions that various pan-HDAC inhibitors have been tested, such as trichostatin A, which showed an increase in the acetylation levels of histones H3 and H4 accompanied by changes in the expression profiles of genes involved in the virulence of the parasite. It is worth mentioning that these virulence factors were also downregulated by RNAi methods, producing a strain that expresses the different VSP without expressing any virulence factor, resulting in a potential preliminary vaccine against this parasite. Lastly, as in the kinetoplastid group, DNA methylation could have a role in regulating LINEs, thereby providing stability to the parasite genome.

In the case of *G. lamblia*, it has been postulated that both HPTM, more specifically acetylated lysines and RNAi, may play important roles in regulating the VSP that is being expressed. Additionally, methyl transferase enzymes are essential for parasite viability in *E. histolytica* and *G. lamblia* by silencing the virulence factor gene *Eh*ap-a (amoebopore) in the first parasite, and in the second, by disrupting its encystment process. Finally, the pan-HDAC inhibitors tested in *G. lamblia* have shown promising inhibitory effects on vital processes of the parasite, together with miR3 that modulates H2A translation, which can be considered epigenetic therapeutic targets.

On the other hand, about *T. gondii* and *T. vaginalis* there is less information on the different epigenetic mechanisms present in each of these parasites. For *T. gondii*, there are few reports of general transcription factors which different epigenetic mechanisms could replace. Additionally, various pan-HDAC inhibitors have been tested in *T. gondii*.

Finally, in *T. vaginalis,* the effect of pan-HDAC inhibitors on gene expression has been described, particularly those regulated by iron, which is involved in important metabolic pathways and virulence. A little information is available about the role played by RNAi in *T. vaginalis*, suggesting that it performs a function in the silencing of transposons, conferring stability to the genome in a similar way to the protists aforementioned. A similar case is the exclusive DNA methylation of *T. vaginalis* (6 mA), which could be associated with silencing transposable elements and possibly gene silencing.

All these mechanisms involve proteins that could be used as potential therapeutic targets, especially those involved in epigenetic processes exclusive to these parasites. Information is still lacking on the epigenetic mechanisms present in lower eukaryotes. However, the evidence obtained to date points to an unprecedented opportunity to use these epigenetic targets for developing lead compounds that can combat these diseases at a lower cost and without the drawbacks of current routinely used drugs.

## Figures and Tables

**Table 1:** Biological activity, dose, and adverse effects of drugs used against Chagas disease.

Drugs	IC_50_ (µM)	Doses	Side Effects
Benznidazole	0.0018 [27]	Oral-Solid: 100 mg tablet; 50 mg tablet (Adults) [30]; 12.5 mg tablets by two months.	Chills, chest pain, fever, severe rash, unusual bleeding or bruising, unusual tiredness or weakness, and painful or difficult urination [31].
Nifurtimox	0.11 [28]	Oral-Solid: 30 mg tablet; 120 mg tablet; 250 mg tablet by two months [32].	Decreased appetite, diarrhea, dizziness, fever, headache, nausea, stomach pain, vomiting, and weight loss [31].

**Table 2:** Biological activity, dose, and adverse effects of drugs used against *T. brucei*.

Drugs	IC_50_ (µM)	Doses	Side Effects
Pentamidine	0.0008 [59]	Parenteral-General injections-IM: 200 mg (as isethionate) powder for injection by one week [32].	Pain and swelling at the injection site, hypotension, vomiting, blood dyscrasias, and renal damage [31].
Suramin	0.005 [60]	Doses are based on body weight and must be determined by a physician within approximately twenty-one days [32].	Nausea, vomiting, diarrhea, headache, skin tingling, and weakness [31].
Melarsoprol	0.0026 [61]	Parenteral-General injections-IV: 3.6% in 5 mL ampoule solution (180 mg of the active compound by ten days) [32].	Convulsions, fever, loss of consciousness, rashes, bloody stools, nausea, and vomiting [31].
Eflornithine	0.026	Parenteral-General injections-IV: 200 mg per mL in 100 mL bottle (hydrochloride) by one week [32].	Sore throat and fever, unusual bleeding or bruising, unusual tiredness or weakness, convulsions (seizures), and loss of hearing [31].
Nifurtimox	0.005	Oral-Solid: 120 mg by ten days [32].	Sore throat and fever, unusual bleeding or bruising, unusual tiredness or weakness, convulsions (seizures), and loss of hearing [31].

**Table 3:** Biological activity, dose, and adverse effects of drugs used against Leishmaniasis.

Drugs	IC_50_	Doses	Side Effects
Glucantime	100 µg/mL [104].	Adults and children, 20 mg per kg of body weight per day injected into a muscle for twenty to twenty-eight 28 days [105].	Fever, arthralgia, myalgia, nausea and vomiting, erythema nodosum, and skin rash [31].
Pentostam	24 µg/mL [106].	Adults 100 mg 3 times a day for three months. Children younger than 16 years of age. Use and dose must be determined by a physician [32].	Black, tarry stools; bleeding of the gums; blindness; blood in the urine or stools; blurred, decreased, or other vision changes; bruising; burning, dry, or itching eyes; chills; and cough [31].
Amphotericin B	0.6 to 0.7 μM [107].	Adults and children: 3 to 6 mg per kilogram of body weight once daily for seven days, injected slowly into a vein [31].	Chills, fever, irregular heartbeat, muscle cramps or pain, unusual tiredness, or weakness [31].
Pentamidine	5.09 µM [108].	A single dose of 7 mg/kg [109].	Decrease in urination, sore throat, fever, unusual bleeding, or bruising [31].
Ketoconazole	2 µM [111].	Oral 600 mg/day for twenty-eight days [110].	Bleeding gums, blood in the urine or stools, blurred vision, burning, itching. Ketoconazole can cause serious harm to the liver that may result in a liver transplant or cause death [31].
Miltefosine	3.8 μM [112].	Oral-Solid: 10 mg; 50 mg for twenty days cutaneous form and twenty-eight to forty days visceral form [32].	Abdominal or stomach pain; bloating or swelling of the face, arms, hands, lower legs, or feet; and possible teratogenicity [31].
Paromomycin	133.8 μM [112].	Parenteral-General injections-IM: 750 mg paromomycin base (as sulfate) for twenty-eight days [32].	Abdominal or stomach cramps, diarrhea, and nausea [31].

**Table 4:** Biological activity, dose, and adverse effects of first- and second-line drugs against amoebiasis.

Drugs	IC_50_ (µM)	Doses	Side Effects
Paromomycin	Not available	Oral-Liquid: 125 mg per 5 mL as sulfate. Oral-Solid: 250 mg as sulfate for five to six days [32].	Abdominal or stomach cramps, diarrhea, and nausea [31].
Metronidazole	0.23 [141]	Oral-Liquid: 200 mg per 5 mL (as benzoate) Oral-Solid: 200 to 500 mg tablet Parenteral-General injections-unspecified: 500 mg in 100 mL vial for five to ten days [32].	Agitation, back pain, blindness, confusion, decreased vision, depression, dizziness, fever, headache, irritability, nausea, seizures, vomiting, and weakness in the arms, hands, legs, or feet [31].
Diiodohydroxyquin	0.082 [142]	Tab 210 mg three times Tab 650 mg three times for ten days [32].	Vomiting, nausea, diarrhea, headaches, skin rashes, and anal pruritis [31].
Diloxanide furoate	Not available	Adults, oral, 500 mg every 8 h for ten days Children > 25 kg, 20 mg/kg per day orally in three separate doses for ten days [32].	Feeling sick (nausea) or being sick (vomiting), loss of appetite, diarrhea, stomach (abdominal) cramps, and wind (flatulence) [31].

**Table 5:** Biological activity, dose, and adverse effects of drugs used against giardiasis.

Drugs	IC_50_ (mg/L)	Doses	Side Effects
Metronidazole	0.21 [177]	15 mg/kg/day (maximum 750 mg/day) orally in three doses for five to ten days [178].	Nausea, abdominal pain, and diarrhea. Rare cases of neurotoxicity, optic neuropathy, peripheral neuropathy, and encephalopathy. Genotoxic effects in animal models are controversial in humans [176].
Tinidazole	0.14 [177]	50 mg/kg (maximum 2 g) orally, single dose [178].	Chest tightness, change in consciousness, cough, loss of consciousness, and trouble breathing [31].
Nitazoxanide	15 nM [177]	7.5 mg/kg orally twice a day for three days [178].	Abdominal or stomach pain, headache, nausea, and urine changes [31].
Albendazole	0.01 [177]	10 to 15 mg/kg (maximum 400 mg) orally once daily for five days [178].	Stomach pain; black, tarry stools; bleeding gums; and blood in the urine or stools [31].
Mebendazole	0.06 [177]	100 mg orally twice a day for three days [178].	Black, tarry stools; chills, convulsions, cough, or hoarseness; dark urine; fever with or without chills; and a general feeling of tiredness or weakness [31].
Furazolidone	0.62 [177]	2 mg/kg (maximum 100 mg) orally, four times daily for seven days [178].	Abdominal or stomach pain, diarrhea, headache, nausea, or vomiting [31].
Secnidazole	0.62 [177]	30 mg/kg (maximum 2 g) orally, single dose [178].	Change in taste, Diarrhea, headache, loss of taste, nausea, stomach pain, and vomiting [31].
Ornidazole	0.12 [177]	20 to 40 mg/kg (maximum 2 g) orally, single dose [178].	Abdominal or stomach pain; anxiety; black, tarry stools; bleeding gums; blood in the urine or stools; blurred vision; body aches; or chest pain [31].

**Table 6:** Biological activity, dose, and adverse effects of drugs used against toxoplasmosis.

Drugs	IC_50_ (µM)	Doses	Side Effects
Spiramycin	Not available	Adults and teenagers—1 to 2 g, two times a day, or 500 mg to 1 g at least four to six weeks after the absence of clinical symptoms [32].	Skin rash and itching, unusual bleeding or bruising [31].
Pyrimethamine	0.08 [201]	Oral-Solid: 25 mg for one to three weeks [32].	Chest pain, dry cough, fever, rapid or trouble breathing, skin rash, unusual tiredness or weakness [31].
Sulfadiazine	100 [202]	100 mg/kg daily, orally, divided twice a day for three to four weeks [32].	Hives; difficulty breathing; swelling of the face, lips, tongue, or throat [31].
Trimethoprim	0.0072 [203]	10 mg/kg/day for four weeks [32].	Skin rash or itching; black tarry stools; blood in urine or stools; bluish fingernails, lips, or skin; changes in facial skin color; and chills [31].
Sulfamethoxazole	Not available	One 800 mg tablet of sulfamethoxazole and 160 mg of trimethoprim or four teaspoonfuls or 20 mL of oral liquid every 12 h for ten to fourteen days [32].	Black, tarry stools; blistering, peeling, or loosening of the skin; chest pain or tightness [31].

**Table 7:** Drugs used for pharmacological treatment of trichomoniasis.

Drugs	IC_50_ (µM)	Doses	Side Effects
Metronidazole	0.068 [222]	Adults: A tablet of 2 g, in a single dose of 1 g twice a day for one day. The capsule 375 mg twice a day for seven days. Children: Use and dose must be determined by a physician [29].	Vomiting, blindness, back pain, burning, numbness, tingling or painful sensations in the hands or feet, dizziness, and drowsiness [31].
Tinidazole	Not available	Adults 2 g given once as a single dose. Children: Use and dose must be determined by a physician [29].	Chest tightness, change in consciousness, loss of consciousness, cough, and trouble breathing [31].

## Data Availability

Data is contained within the article.
